# A Study on the Morphometry of a Medial Meniscus in the Knee Joint of Human Cadavers in the South Indian Population

**DOI:** 10.7759/cureus.42753

**Published:** 2023-07-31

**Authors:** Saravanan Subramanian, Arun Prasath Balakrishnan

**Affiliations:** 1 Department of Anatomy, Government Villupuram Medical College, Villupuram, IND

**Keywords:** arthroscopy knee, prostheses, medial meniscus, morphometry, knee joint

## Abstract

Introduction

The knee joint is a complex system containing various hard and soft tissue components necessary for functioning in a coordinated manner. The menisci help to deepen the tibial plateau. Knowledge of the dimension of menisci in the knee joint is of paramount importance in arthroscopic surgery and the management of injuries due to sports or degeneration. The present study aims to describe the morphometric data of the medial meniscus and document the morphometric variation in the medial menisci.

Methodology

This study was conducted in the department of anatomy in two medical colleges under MGR University by measuring the dimensions of 100 medial menisci taken from 50 formalin-fixed embalmed cadavers. The width and thickness of the medial menisci were measured using digital vernier calipers. The outer and inner circumferences were measured using a measuring tape, non-elastic threads, and metallic pins. The area of the medial meniscus and the tibial plateau was measured by counting the small squares present in the circumference of the menisci drawn over the graph paper. The weight of the medial menisci was measured using the electronic weigh scale.

Results

The widest part of the medial meniscus was the posterior one-third, and the narrowest part was the anterior one-third. The thickest part was the middle one-third, followed by the anterior one-third. The average inner and outer circumferences of the menisci were 6.25 cm and 10.05 cm, respectively. The medial meniscus covers more than half of the area of the tibial plateau.

Conclusion

The present study provides a good understanding of the morphometric features of the medial menisci and will be of great help for managing knee joint pathologies and designing prostheses.

## Introduction

The medial meniscus forms the main supporting soft tissue between the osseous portion of the femur and tibia, which helps in performing functions like stabilization, rotation, and weight transmission in the knee joint [1]. The medial and lateral menisci are a pair of semilunar cartilages present between the tibial plateau and femoral condyles [2]. The structure of the knee joint during torsion is well maintained by these cartilages [3]. Variations in the form, contour, and insertion of the menisci can determine the mechanism and kind of injury [1]. These menisci have an avascular inner zone and a vascular outer zone. During trauma, healing is delayed in the avascular zone, whereas healing is better in the vascular zone if removed surgically [4]. Injury to the meniscus is on the rise because of increased BMI due to a sedentary lifestyle [5]. For transplantation and repair of menisci, the morphometric value is an important requirement. The data available on the morphology and morphometry of the medial menisci are scarce. Hence, the current study was conducted to measure morphometric parameters like width, thickness, outer circumference, inner circumference, area of the medial meniscus, the ratio of the area of the medial meniscus to the area of the tibial plateau, and weight of the medial meniscus.

## Materials and methods

This study was conducted in the department of anatomy in two medical colleges under MGR University between 2014 and 2018 by measuring 100 medial menisci taken randomly from 50 formalin-fixed embalmed cadavers aged between 20 and 80 years. Of the 50 embalmed cadavers, 25 were males and 25 were females. In this study, knee joints with musculoskeletal anomalies, degenerated and traumatized menisci, were excluded. The menisci were dissected first by making a longitudinal incision in the anterior side of the joint capsule. The patellar ligament and collateral ligaments were incised transversely. After removing the intra-articular ligament and the joint capsule, the menisci were exposed. The menisci were removed with utmost caution and detached from the femoral condyles and tibial plateau [6].

**Figure 1:**
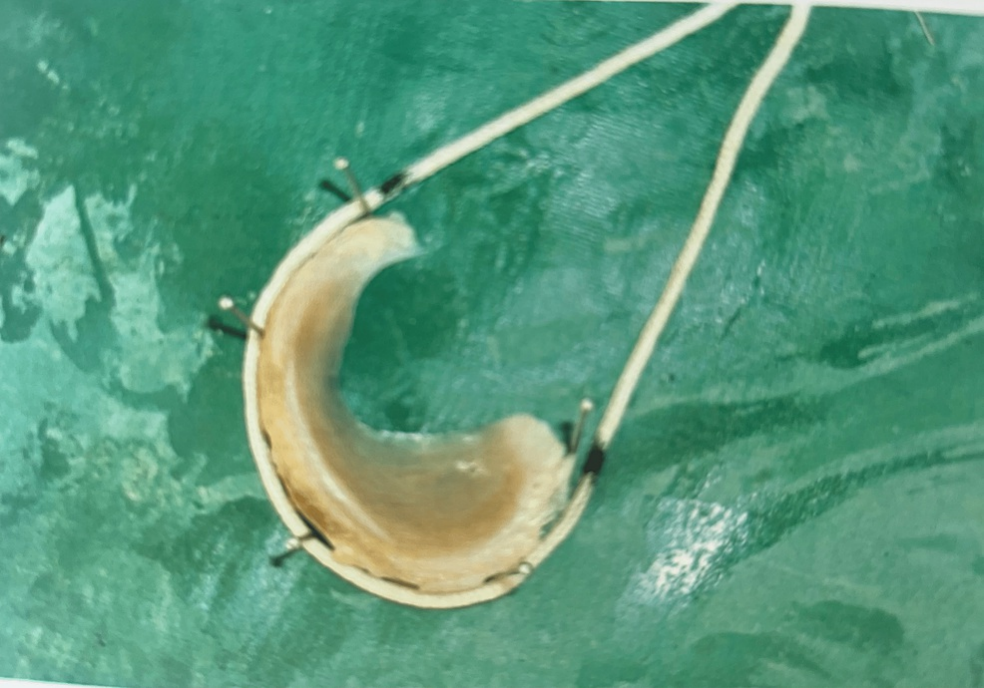
Outer circumference of the meniscus measured using a non-elastic thread

The data were recorded systematically. The peripheral length of the menisci was measured using a non-elastic cotton thread [4]. The metallic pins were placed along the periphery of the meniscus, and the thread was placed along the periphery. The anterior-most point and the posterior-most point were marked. The distance between the two points gave the outer peripheral length, which is the outer circumference (Figure 1) [6].

The metallic pins were then placed on the inner border. The anteriormost point and the posteriormost point were marked. The length of the thread from the anteriormost point to the posteriormost point gave the inner circumference. The peripheral length obtained by using the thread was divided into three equal parts. The portion of the menisci corresponding to these parts was termed the anterior one-third, middle one-third, and posterior one-third, respectively.

**Figure 2:**
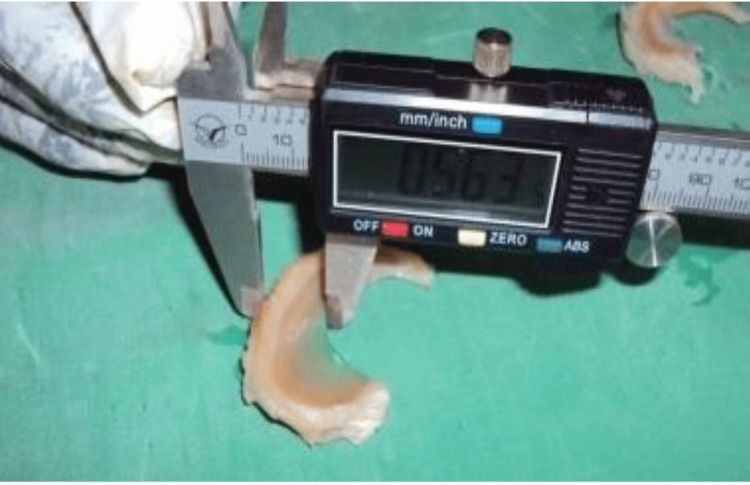
Width of the meniscus measured using a vernier caliper

The width and thickness of the medial menisci were measured at the midpoint of the anterior one-third, middle one-third, and posterior one-third, respectively, using a vernier caliper (Figure 2) [5].

**Figure 3:**
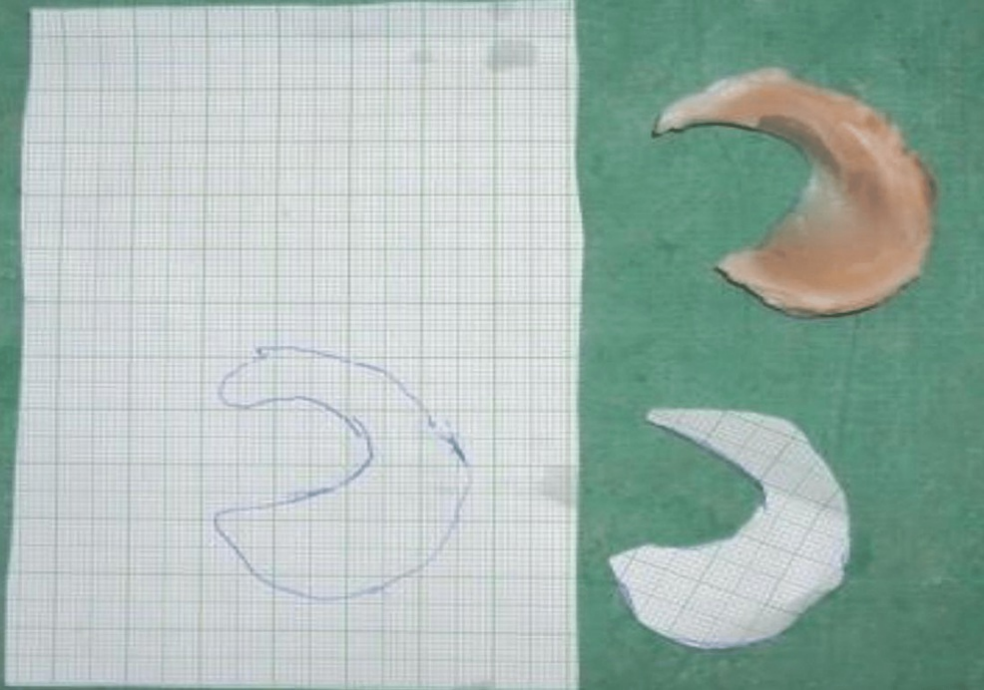
Area of the meniscus measured using graph paper

The area of the medial menisci and tibial plateau was obtained by measuring the contour using litmus paper, which was then drawn on graph paper. The number of small squares within the contour drawn on the graph paper gave the area of the medial meniscus and tibial plateau in mm2 (Figure 3) [3].

The weight of the medial meniscus was measured using an electronic weighing machine.

The width and thickness were measured in millimeters with an accuracy of 0.01 mm. The outer circumference and inner circumference were measured in centimeters with 0.01 mm accuracy. The area was measured in the unit of millimeter squares with an accuracy of 0.01 mm2. The weight is measured in milligrams with an accuracy of 0.01 mg. The data were analyzed using SPSS Statistics version 22.0 (IBM Corp. Released 2013. IBM SPSS Statistics for Windows, Version 22.0. Armonk, NY: IBM Corp.) and recorded.

## Results

The present study was conducted to measure the dimensions of the medial meniscus using morphometric parameters like width, thickness, outer circumference, inner circumference, the ratio of the area of the medial meniscus to the area of the tibial plateau, and weight. The measured data were systematically arranged, and the values were tabulated and analyzed (Table 1).

**Table 1:** Width and thickness of medial menisci (n= 100)

	Width			Thickness		
Medial meniscus	Anterior 1/3	Middle 1/3	Posterior 1/3	Anterior 1/3	Middle 1/3	Posterior 1/3
Minimum	9.5 mm	9.6 mm	14.5 mm	5.91 mm	6.08 mm	5.06 mm
Maximum	10.3 mm	10.6 mm	15.5 mm	6.34 mm	6.54 mm	5.51 mm
Average	9.8 mm	9.9 mm	15.0 mm	6.14 mm	6.33 mm	5.29 mm
SD	0.17	0.22	0.27	0.109	0.124	0.107

The average width of the anterior one-third was 9.8 ± 0.17 mm, the middle one-third was 9.9 ± 0.22 mm, and the posterior one-third was 15.0 ± 0.27mm. The average thickness of the anterior one-third was 6.14 ± 0.109 mm, the middle one-third was 6.33 ± 0.124 mm, and the posterior one-third was 5.29 ± 0.107 mm (Table 2).

**Table 2:** Outer circumference, inner circumference, weight, surface area, ratio of medial meniscus area to tibial plateau area (n=100)

Statistical data	Outer circumference	Inner circumference	Weight	Surface area	The ratio of the medial meniscus area to the tibial plateau area
Minimum	9.82 cm	5.98 cm	1.63 g	412 mm2	47.00
Maximum	10.28 cm	6.44 cm	2.16 g	508 mm2	65.51
Average	10.05 cm	6.25 cm	1.88 g	466 mm2	54.56
SD	0.12	0.12	0.135	23.94	4.06

The average outer circumference of the medial meniscus was 10.05 ± 0.12 cm. The average inner circumference of the medial meniscus was 6.25 ± 0.12 cm. The average weight of the medial meniscus was 1.88± 0.135 g. The average surface area was 466 ± 23.94 mm2, and the ratio of the area of the medial meniscus to the area of the tibial plateau was 54.56 ±.4.06.

## Discussion

The intra-articular structures of knee joints have been extensively studied in recent years due to advancements in arthroscopic procedures [5]. The morphometric data are important variables in the clinical diagnosis and surgical procedures of the knee joint [7].

The present study shows that the widest part is the posterior one-third, followed by the middle one-third and the anterior one-third. From this, we can infer that the widest part is the posterior one-third and the narrowest part is the anterior one-third. The results obtained were consistent with the studies conducted by Amandeep et al. [8], Braz et al. [9], Chintan et al. [3], Almedia et al. [1], and Chaware et al. [10]. The wider area of the meniscus allows more action on the femoral condyles and is more prone to injury compared to the narrow part [5]. Literature reveals the anterior one-third of the menisci is least prone to injury [1,3]. This fact is substantiated in the present study.

The present study shows the thickness is 6.14 mm for the anterior one-third, 6.33 mm for the middle one-third, and 5.29 mm for the posterior one-third. The present study shows the thickest part is the middle one-third and the thinnest is the posterior one-third. The present study confirms other studies like Amandeep et al. [8], Braz et al. [9], and Hathilia et al. [11]. The kind of injury, location, and type of injury were determined by the width and thickness of the medial meniscus [6]. It is documented that there is an inverse relationship between the width and thickness of the menisci. This correlates with the fact that menisci with greater width and thinner menisci are easily injured. The present study justifies this finding (Table 3) [5].

**Table 3:** Comparison of width and thickness with previous studies

		Width			Thickness		
Sl no	Name of study	Anterior 1/3	Middle 1/3	Posterior 1/3	Anterior 1/3	Middle 1/3	Posterior 1/3
1	Almedia et al. (2004) [1]	9.02 mm	12.16 mm	17.37 mm	5.92 mm	5.31 mm	5.91 mm
2	Braz et al. (2010) [9]	7.68 mm	9.32 mm	14.96 mm	6.17 mm	6.31 mm	5.18 mm
3	Amandeep et al. (2013) [8]	9 mm(rt), 10 mm(lt)	10 mm(rt), 11 mm(lt)	14 mm(rt), 15 mm(lt)	6.17 mm	6.31 mm	5.18 mm
4	Chintan et al. (2014) [3]	8.78 mm	12.08 mm	16.46 mm	5.82 mm	5.64 mm	5.86 mm
5	Chaware et al. (2023) [10]	7.05 mm	8.12 mm	13.78 mm	4.45 mm	5.50 mm	5.95 mm
6	Present study	9.8 mm	9.9 mm	15.0 mm	6.14 mm	6.33 mm	5.29 mm

The menisci cover from half to two-thirds of the articular surface of the corresponding tibial plateau [1,3]. In the present study, the average ratio of the area of the medial meniscus to the area of the tibial plateau is 54.56, which implies that the medial menisci cover more than half of the tibial plateau. This justifies the increased incidence of meniscus injuries because of the increased action of the femoral condyles [1]. The results obtained were similar to those of other studies like Messener et al. [2] and Chintan et al. [3].

In the present study, the average outer circumference is 10.05 cm. This finding is consistent with studies by Amandeep et al. [8], Braz et al. [9], and Chintan et al. [3]. In the present study, the average inner circumference was 6.25 cm, which is similar to results obtained in studies by Amandeep et al. [8] and Narayana Rao et al. [5]. In the present study, the average weight was 1.84 g, which is the same as the result obtained in the study by Amandeep et al. [8].

All these parameters should be kept in mind while managing knee joint pathologies and while manufacturing prostheses for knee joints. The study limitation is that the medial meniscus is a curved non-linear structure, for which the technique for measuring the linear structure was followed (Table 4) [12].

**Table 4:** Outer circumference, inner circumference, weight, surface area, and the ratio of the area of the medial meniscus to the tibial plateau comparison with previous studies

Sl no	Name of the study	Outer circumference	Inner circumference	Weight	Medial meniscus area	Ratio of medial meniscus area to tibial plateau area
1	Braz et al. (2010) [9]	9.8 cm	----	-----	---	---
2	Amandeep et al. (2013) [8]	9.2c m[rt], 9.7 cm[lt]	6.2 cm[rt], 6.4 cm[lt]	1.8 g[rt], 1.87 g[lt]	---	---
3	Narayana rao et al. (2014) [5]	8.64 cm	5.1 cm	----	---	---
4	Chintan et al. (2014) [3]	10.46 cm	----	-----	450.88 mm2	72.6
5	Present study	10.05 cm	6.25 cm	1.86 g	466 mm2	54.56

## Conclusions

This study provides the morphometric parameters that act as a useful tool for the diagnosis and management of injuries in knee joints due to trauma or degeneration. The above parameters can further be evaluated by radiological methods and can be correlated with actual parameters. The present study shows the posterior one-third is the widest and thinnest, the middle one-third is the thickest, and the anterior one-third is the narrowest part of the menisci. This justifies the increased incidence of injury in the posterior one-third. The results are in unison with other studies and provide comprehensive morphometric features for designing artificial meniscal prostheses, for procedures like arthroscopy and advanced knee surgeries.
